# Factors Associated with the Early Introduction of Complementary Feeding in Saudi Arabia

**DOI:** 10.3390/ijerph13070702

**Published:** 2016-07-12

**Authors:** Riyadh A. Alzaheb

**Affiliations:** Department of Clinical Nutrition, Faculty of Applied Medical Sciences, University of Tabuk, Tabuk 71491, Saudi Arabia; ralzaheb@ut.edu.sa; Tel.: +966-144-562-723

**Keywords:** the introduction of complementary feeding, risk factors, Saudi Arabia

## Abstract

Mothers’ instigation of complementary feeding before their infant reaches 6 months old risks shortening their breastfeeding duration, and high morbidity and mortality for their child. Complementary feeding practices require further investigation in Saudi Arabia. The present study aims to evaluate complementary feeding practices, and to establish which factors are associated with the early introduction of complementary feeding in the Saudi Arabian context. Cross-sectional research was conducted with 632 mothers of infants aged between 4 and 24 months attending five primary health care centers (PHCCs) between July and December 2015 in Saudi Arabia. Data on participants’ socio-demographic characteristics and complementary feeding practices were collected via structured questionnaires. A regression analysis identified the factors associated with the early introduction of solid foods, defined as before 17 weeks. 62.5% of the study’s infants received solid foods before reaching 17 weeks old. The maternal factors at higher risk of early introduction of solids were: younger age; Saudi nationality; shorter education; employment within 6 months post-birth; caesareans; not breastfeeding fully for six weeks post-birth, and living in low-income households. Complementary feeding prior to 6 months postpartum was common in Saudi Arabia. Public health interventions are needed to reduce early complementary feeding, focusing on mothers at highest risk of giving solids too early.

## 1. Introduction

The stage of life beginning at birth and lasting until an infant reaches two years of age is known as the “critical window” in terms of encouraging optimal health, growth and cognitive development [1]. Suitable feeding practices are therefore vital to the healthy development and growth of the infant during this stage of their early life [2]. Addressing this in 2001, the World Health Organization (WHO) set out the recommendation that mothers in both developing and developed countries should exclusively breastfeed their infants for the first 6 months of their lives, and then ensure that they receive adequately safe and nutritious solid foods alongside continued breastfeeding until they reach the age of 2 years or more [3,4]. The research evidence to date supports the WHO’s recommendation that complementary foods should not be introduced before infants reach 6 months of age [5,6].

The appropriate introduction and use of complementary feeding is a key determinant influencing the healthy development and growth of infants [7]. The early introduction of complementary foods (i.e., before the infant reaches six months of age) may have the effect of replacing breast milk and halting breastfeeding altogether at too early a stage [8,9]. The early introduction of solids as alternatives to breast milk also means that infants are more likely to be exposed to microbial contaminated foods and fluids; this is especially the case in developing countries [2]. A lower level of breast milk consumption and/or contaminated foods causing infections may result in infants facing malnourishment and/or experiencing poor growth [10]. A study has also found that young infants under six months of age are not yet ready physiologically to receive complementary food as their neurodevelopment, gastrointestinal systems, and kidneys are still underdeveloped [8]. A systematic review in 2013 found evidence suggesting that the very early introduction of solid foods (at or before an infant reaches 4 months of age), instead of at 4–6 months or > 6 months, may result in an increased risk of infants becoming overweight in childhood [11].

Despite the WHO’s recommendations and the clear evidence of several adverse effects on infants’ health which can potentially be associated with it, the early instigation of complementary feeding remains common across both developed and developing countries. A European multicenter study covering five countries: Belgium, Germany, Italy, Poland, and Spain, found that approximately 25% of infants began weaning before reaching the age of 4 months, and that by the age of 6 months, at least 90% of them had consumed solid foods [12]. Previous research studies into complementary feeding in the Middle Eastern context have also found that contemporary practices do not follow the global recommendations set out by the WHO; for example, in Iraq, the United Arab Emirates and Lebanon, 78.6%, 70% and 52.9% of infants respectively are given complementary foods while they are still between 4 and 6 months old [13].

Various factors have been associated in different research settings with mothers’ timing of the introduction of complementary feeding. Studies in western countries have associated the early introduction of complementary feeding with specific maternal characteristics, including their age, education, occupation and marital status; socioeconomic status; pre-pregnant body mass index (BMI) and location of residence; as well as infant characteristics such as birth weight, delivery method, order of birth, and pacifier use [9,14,15]. In comparison to the western research, less is known about the factors influencing the age at which solid foods are introduced by Middle Eastern mothers [13,14].

The Kingdom of Saudi Arabia (KSA) has a total population of 27 million people, and covers two million square kilometers in the Middle East. Despite the limited evidence produced to date on breastfeeding in Saudi Arabia [16], complementary feeding practices have not been studied in the country. The lack of data on complementary feeding practices focuses research attention onto the information gaps relating to the factors which lead to unsuitable complementary feeding practices (such as its early introduction) in the KSA, and onto the information which is needed to develop effective and lasting community-level health interventions designed to encourage better complementary feeding practices.

The aims of the present research are therefore to evaluate complementary feeding practices, and to identify the primary factors associated with the early introduction of complementary feeding. The results arising from the research can potentially be used to form the evidence required to design effective strategies encouraging appropriate complementary feeding in Saudi Arabia. Effective complementary feeding interventions do not have a fixed set of component parts, because their target populations have varied circumstances and needs [17].

## 2. Methods

This research performed a cross-sectional survey in Tabuk city, Saudi Arabia, between July and December 2015, which gathered data on feeding practices among mothers of infants aged 4–24 months who were attending five of the city’s 21 primary health care centers (PHCCs). Each PHCC selected for the survey was in each of the five geographic regions of Tabuk (north, south, east, west and central), and was selected via random systematic sampling stratification by population density, geographical location and socioeconomic status. The five PHCCs used in this study were each posted a letter from the regional health administrator with an attached factsheet outlining the objectives of the research and requesting the assistance of trained female nurses with the completion of questionnaires.

The sample size for the research was calculated by utilizing the Epi-Info statistical program and using the total of 12,760 live births registered in Tabuk in 2014, with an assumption of a 50% prevalence of the early introduction of complementary feeding (<17 weeks of age), a 95% confidence level, and a confidence limit of 5%. The resulting sample size was 560 infants, which was then approximated to 600 infants.

Mothers with healthy infants of 4–24 months of age who were attending each of the selected PHCCs were personally approached when they arrived for scheduled health examinations, and were asked to attend an interview after being given a full orientation regarding the research. If they accepted the invitation to be interviewed, they were given an information sheet and asked to sign a consent form confirming their participation in the research. To be more precise, in the case of a mother who had more than one infant under two years of age, the data she provided related to her youngest child.

The survey was used to collect the data required by the research during the PHCC’s business hours, so the mothers invited for interview (so that their questionnaires could be completed) were also able to keep their health appointments. Almost all mothers in Saudi Arabia with children under five years of age visit PHCCs for their infants’ vaccinations [18].

### 2.1. Data Collection

In all, 664 eligible mothers were invited to participate in this research, of whom 632 completed the structured questionnaire, meaning that the response rate was 95.2%. The participating mothers at each PHCC gave their responses to the structured questionnaires with the support of trained, Arabic-speaking, female nurses. These surveys collected data relating to the mothers’ past and present infant feeding practices, including their breastfeeding duration and practices and, use of formula milk, and the timing of their introduction of solid foods to their infants. The survey also gathered data on each mother’s age, education duration, employment status, nationality, family income (in Saudi Riyals), number of children, and infant delivery mode; as well as the infant’s father’s age, education, employment status and nationality, and the infant’s age, sex, and birth weight.

Prior to administering the questionnaires to the full sample of participants, a pilot study was carried out with 25 participating mothers to test the questionnaire design, in order to make any alterations required after feedback was gathered and evaluated. The pilot study established that questionnaire completion took an average of 15 min. The participants in the pilot study were not included in the final study sample.

### 2.2. Ethical Approval

This research sought and gained ethical approval from the University of Tabuk’s Committee of Research Ethics (HAP-07-TU-001).

### 2.3. Data Analysis

The data gathered from the surveys were inputted and analysed with the use of the Statistical Package for the Social Sciences (SPSS) program, version 21.0 (IBM, New York, NY, USA). It should be noted that although at present the WHO’s recommended age at which complementary foods should be introduced is 6 months [4], in practice it is relatively rare in Middle Eastern countries to introduce solids on or after the infant reaches 6 months of age [13,14]. The analysis in this research therefore uses the dependent variable “the early introduction of complementary feeding”, which is defined here as introduction before the infant has reached 17 weeks. This reflects the European Society’s guidance that complementary foods should not be given to infants before they reach 17 weeks of age [19]. The definition used in the present study for the early introduction of complementary feeding is the same as that used by prior studies with similar objectives which have been carried out both in Middle Eastern countries (for example, in Kuwait) [14], and also in Western countries [20,21].

The dependent variable mentioned above, “the early introduction of complementary feeding”, was constructed from one of the questionnaire’s questions which asked the participants to state the point in time when their infants started to receive solid foods (i.e., complementary foods) at least twice daily over a period of several weeks. In this study, solid foods were defined as pureed fruits, baby cereals, etc., rather than any form of milk or other drinks. A binary dependent variable was then created, with two categories: <17 weeks for the early introduction of complementary feeding, and ≥17 weeks for the introduction of complementary feeding at an acceptable time.

Previous studies have identified associations between various maternal and infant characteristics with the age at which the introduction of complementary feeding occurs, and these were therefore investigated by the present research. The maternal variables included the mothers’ age (<25, 25–35, >35 years), nationality (Saudi vs. Non-Saudi), years of education (<12 vs. ≥12 years), employment status in the first 6 months postpartum (in employment vs. not in employment), household income (<5000, 5000–10,000, 10,001–15,000, >15,000 Saudi Riyals), number of children (1 vs. 2+ children), and mode of delivery (Caesarean vs. Vaginal). Meanwhile, the infant related variables included their gender, birth weight (<2.5 kg vs. ≥2.5 kg) and their exclusive breastfeeding status at the age of 6 weeks.

The associations between each of the potential explanatory variables and the early complementary feeding variable were initially assessed one by one using binary logistic regressions. All the variables with a *p* value < 0.2 were then included in multivariate logistic regression in order to identify those which independently predicted the early introduction of complementary feeding. Odds ratios (OR) and their 95% confidence intervals (CI) were produced. The statistical significance level selected for the study was *p* ≤ 0.05.

## 3. Results

Table 1 outlines the relevant characteristics of the mothers and families in the study sample. Of the 632 mothers who participated, a large majority (89.6%) held Saudi nationality, and approximately 60% of the total participants were aged between 25 and 35 years. Just over half of the participating mothers (51.4%) had attended school for less than 12 years. With regard to the mothers’ employment status, 20.9% were working in the first 6 months after giving birth to their child. 45% of mothers lived in households with incomes of between 5000 and 10,000 Saudi Riyals per month. Further, 64.2% of the mothers were multiparous, and 74.8% had delivered their infants vaginally. The majority of the fathers (57.3%) in the sample were also aged between 25 and 35 years. A large majority held Saudi nationality (89.9) and a majority (59.8%) had attended school for less than 12 years. 91.0% of the fathers in the sample were in employment, which was a considerably higher proportion than among the mothers. Lastly, the ratio of infant gender was close to 1:1, and 90.2% of infants had a birth weight ≥2.5 kg.

The data gathered in this study relating to complementary feeding practices are displayed in Table 2. A high proportion (62.5%) of the infants in the study sample (*n* = 632) were given complementary foods prior to reaching 17 weeks old. The participating mothers gave several reasons for feeding their infants with solid foods before the 17 week mark, the main ones being their baby’s hunger (51.4%) and that they believed their baby was old enough to receive solids (26.6%). Baby cereal was the most frequently given solid, having been used in over 60% of instances for the two age groups describing the introduction of complementary foods (<17 weeks and ≥17 weeks). Of the participating mothers whose infants received complementary foods <17 weeks of age, 75.4% reported difficulties in introducing the new foods. In contrast, only 48.5% of mothers whose infants received complementary foods ≥17 weeks of age reported difficulties. The most common explanations given by the mothers for the difficulties they experienced in the introduction of solid foods to their infants included that the infants preferred drinks over food, or only accepted some solid foods and not others. As the table also shows, 36.5% of the infants who were given complementary foods <17 weeks of age needed more than 30 min to finish their meals, while only 23.6% of infants who were only fed complementary foods ≥17 weeks of age needed the same length of time. It should be noted that 31.1% of infants who were fed solid foods <17 weeks of age received food with added salt, but this proportion almost doubled (60.3%) among infants who received complementary foods ≥17 weeks of age. Further, 22% of mothers whose infants received solid foods <17 weeks of age added sugar to their infant foods, rising to 33.3% for those fed solid foods ≥17 weeks of age. The most commonly avoided food group for both age groups in the introduction of complementary foods was found to be nuts (100%), followed by honey, salad vegetables, and fresh fruit. The most popular explanations for avoiding specific types of foods for infants given solid foods <17 weeks of age were that the infant disliked them (32.2%), health reasons (23%) and the possibility of choking (22.5%), while the mothers of those fed solid foods ≥17 weeks of age most commonly cited that the infant disliked them (47.3%), that the food was not cooked at home (17.3%) and that there was the possibility of choking (13.9%).

Table 3 contains the outcomes of the univariate and multivariate logistic regression analyses used to reveal the variables most likely to influence the early introduction of complementary feeding. After carrying out the univariate logistic regression, several variables were identified as having a significant association with the early introduction of complementary feeding: the mother’s age, her nationality, the duration of her education, employment status within the 6 months after giving birth, the mode of delivery, her infant’s weight at birth, and whether or not she was exclusively breastfeeding at 6 weeks postpartum.

After adjustment, the significant factors were shown to be younger mothers (OR = 2.37; 95% CI: 1.21, 4.62 for mothers aged <25 years and OR = 5.27; 95% CI: 2.82, 9.86 for mothers aged 25–35 years); mothers with fewer years of education (<12 years of schooling) (OR = 3.88; 95% CI: 2.54, 5.93); mothers holding Saudi nationality (OR = 2.45; 95% CI: 1.23, 4.87); mothers who were employed within 6 months of giving birth (OR = 6.39; 95% CI: 3.53, 11.58); mothers living in households with incomes of between 5000 and 10,000 Saudi Riyals per month (OR = 3.75; 95% CI: 1.53, 9.17); mothers who gave birth by caesarean section (OR = 4.63; 95% CI: 2.51, 8.52); and mothers who were not exclusively breastfeeding at six weeks after giving birth (OR = 3.27; 95% CI: 2.15, 4.96).

## 4. Discussion

This study has shown that a high proportion (62.5%) of infants in Saudi Arabia are given solid foods prior to reaching 17 weeks of age, a finding which implies a general failure in the country to follow the WHO’s recommendations on the feeding of infants. Higher rates of early introduction of complementary feeding have also been found in various previous research studies in the Middle East context; for example, 65% of infants were reported to have received solid foods at the age of 3 months in the United Arab Emirates [22]; 41.6% of infants received solid foods by the age of 4 months in Lebanon [23]; 35% of infants received solid foods by the age of 5 months in Turkey [24]; and 30.4% of infants received solid foods by the age of 17 weeks in Kuwait [14]. The figures reported by these past studies are similar to the findings of various prior studies in the contexts of industrialised populations, such as 70% of infants in Canada, 63% in Finland, 45% in New Zealand, and 40% in Australia reportedly receiving complementary feeding before reaching 4 months of age [21].

In this research, the participating mothers gave two main reasons for introducing complementary feeding to their infants prior to reaching 17 weeks of age: that their baby was hungry (51.4%), and the mother’s belief that their child was old enough to be given solid foods (26.6%). A prior review examining complementary feeding in the Middle Eastern context came to similar conclusions, finding that in several countries in the region the common reasons given for the early introduction of solids were that breast milk is “nutritionally insufficient” by itself to feed an infant at three or four months old, and simply that “the child is old enough” [13]. Further, in a study in Australia, a developed country, the main reasons mothers put forward for introducing complementary feeding prior to 17 weeks were their infant’s hunger; that their baby was well-developed for their age; and that they believed that their child was sufficiently old to be given solids [21]. Across the studies mentioned here, then, there is evidence of a widespread perception that breast milk on its own is nutritionally insufficient, with the consequence that many mothers introduce their infant to solids at very young ages. Improved education is therefore needed to improve awareness of the advantages of exclusive breastfeeding, including information on the potentially negative effects of introducing complementary foods at too early a stage in an infant’s life.

The current research has identified associations between seven factors and the early introduction of complementary feeding (<17 weeks). Younger mothers were observed to be the likeliest to introduce early complementary feeding. This finding is in line with a previous systematic review of infant feeding practices in developing countries, which found that a young maternal age was a determinant of early complementary feeding [25]. On the other hand, a pair of recent studies in the Middle Eastern context have each examined the factors associated with early complementary feeding, in the contexts of Kuwait [14] and the United Arab Emirates [22] respectively, without finding any association between the mother’s age and early complementary feeding. Turning to research in the context of developed countries, several prior research studies have found that younger mothers have a higher likelihood of early complementary feeding [21]. The findings of a recent study in the United States context were in agreement, having identified a positive association between the timing of the introduction of solid foods and the age of the mother [26].

Further, the present research has established that mothers who were not fully breastfeeding their infants at six weeks after giving birth had a 3.27 times greater likelihood of introducing complementary feeding early than those who were breastfeeding at this stage. However, a study in the Kuwaiti context offered the contrasting findings that mothers breastfeeding at 6 weeks after giving birth had a higher likelihood of introducing solid foods to their infant prior to 17 weeks than mothers who were exclusively giving their infants formula milk at this stage [14]. In developed counties, a systematic review examining the factors influencing the early introduction of complementary food found strong indications that non-breastfeeding mothers have a higher likelihood of early weaning [25]. This finding is in line with recent research in the contexts of Canada [27], Denmark [9], the United Kingdom [28] and the United States [26], each of which have found a positive association between early weaning and the short duration of breastfeeding. Strategies which have been designed to encourage longer breastfeeding durations may therefore help to lower the proportion of mothers who wean their infants early in the initial 6 months of their lives.

The present research has also found caesarean section delivery and living in low-income households to be influencing factors with regard to the early introduction of complementary feeding. With regard to the former factor, caesarean section delivery, no significant relationship of this kind was found by studies in the United Arab Emirates [22], Lebanon [23], or China [2]. However, a systematic review of 53 studies from 33 different countries did reveal a link between caesarean births and lower rates of breastfeeding [29]. This possible negative association found in some studies between caesarean delivery and breastfeeding is also, in turn, connected with the association between the shorter duration of breastfeeding and the early introduction of solid foods. The association between the latter factor, household income, and the early introduction of complementary feeding has not been evaluated by any of the prior studies which have investigated the determinants of early complementary feeding, either in the context of Middle Eastern countries [14,23], or in Western countries [14,20,21]. One study in the context of China aimed to investigate the determinants associated with the early introduction of complementary feeding, but no association was found between the early introduction and low-income households [2]. In the Saudi Arabian context, this possible association is still in need of exploration by future studies.

This study also found mothers with fewer than 12 years of education and mothers employed within 6 months of giving birth to represent variables which could be associated with early complementary feeding. With regard to the former factor, a recent study in the Kuwaiti context did not find any association between mothers’ education levels and the timing of their introduction of complementary foods [14], however this contradicted two earlier Kuwaiti studies [30,31] which had found a strong association between the introduction of complementary foods and maternal levels of education, and more specifically that illiterate mothers had a higher likelihood than women who had attended university of introducing complementary foods when their infants were older. The recent Kuwaiti study’s inability to confirm an association with maternal education as other studies had previously done may have been caused by its weak statistical power [14]. In the context of developed countries, a systematic review [25] and a recent research paper [26] have each found associations between low levels of maternal education and mothers’ early instigation of complementary feeding. Moving on to maternal employment, the present study is in line with previous studies in finding this factor to be negatively associated with early complementary feeding in the context of developing countries [22,23,32] and in developed countries [2], where early complementary feeding was found to be more likely among working mothers. In order to decrease the proportion of mothers starting complementary feeding early, new mothers (especially those with low levels of education) need to be provided with sufficient accessible information, and programs which can support working mothers must be implemented.

One of the key findings emerging from this study is that mothers of Saudi Arabian nationality had a higher likelihood of introducing complementary feeding early than mothers of other nationalities living in Saudi Arabia. This finding is in accordance with a previous study in the Kuwaiti context which concluded that Kuwaiti mothers were more likely to introduce solids within the first 17 weeks than non-Kuwaiti Arab mothers [14]. Scott et al. offered the possible explanation that non-Kuwaiti Arab women are usually the wives of immigrant manual workers from poorer Arab states, so it is possible that they put off the introduction of solid foods in order to save money. This possibility may also hold true in relation to the present study, as Saudi Arabia and Kuwait share many similarities in terms of their culture, economy, and hosting of guest workers from other Arab states. Prior to Scott’s study in Kuwait, a review of infant-feeding practices in the context of developing countries by Arabi et al. had found that caregivers in countries with a high socio-economic status were more likely to report that their infants were being given solid foods than those living in countries with a lower socioeconomic status [33]. The possible associations between ethnicity/nationality and the timing of the introduction of complementary feeding has also been investigated in other regions of the world [24,34,35]. For example, an Irish study found that a higher percentage of mothers of Irish nationality introduced solid foods early than mothers of other nationalities or ethnicities did [20].

When the findings of this research are assessed and interpreted, several limitations must be borne in mind. One key limitation is an unavoidable recall bias which arises because of the data collection’s inevitably retrospective nature, and which can result in participants over- or under-estimating the complementary feeding practices they have implemented in the past. A further inherent limitation is that the present research only involved mothers presenting at government-run PHCCs, which meant that mothers attending other health centers (such as privately-run clinics) were unavailable for inclusion in the sample; this is a limitation for the study because mothers attending private clinics may be members of other socioeconomic classes, so their patterns of complementary feeding and the influences upon it may differ. Also, epidemiological studies such as the present one cannot claim to establish causality, although they may indicate possible associations.

## 5. Conclusions

The research presented here has reached some important conclusions regarding complementary feeding practices in Saudi Arabia, and identified the most influential factors associated with the early (i.e., before 17 weeks) introduction of complementary feeding by mothers in the country. Nearly two thirds of the sampled infants had received solid foods before reaching 17 weeks of age; this is a relatively higher proportion than has previously been reported by similar studies in other countries. The maternal factors with a higher risk of introducing solid foods early are: younger ages; fewer years of education; Saudi nationality; employment within 6 months after giving birth; living in low-income households, caesarean section births; and not fully breastfeeding six weeks post-birth. It is therefore apparent that further public health interventions are urgently required in Saudi Arabia in order to discourage the early introduction of complementary feeding, with a focus placed on these highest-risk groups of mothers. Some of the identified risk factors are potentially modifiable, and thus represent opportunities to encourage optimal timing of mothers’ introduction of solid foods by supporting exclusive breastfeeding in the first 6 months of life, and educating new mothers about the possible negative effects of introducing solid foods too early in their infant’s life.

## Figures and Tables

**Table 1:** Characteristics of the study population (*n* = 632).

Characteristic	Number (*n*)	Percentage (%)
**Mothers**		
Age		
<25 yrs	152	24.1
25–35 yrs	385	60.9
>35 yrs	95	15.0
Education duration		
<12 yrs	325	51.4
≥12 yrs	307	48.6
Nationality		
Saudi	566	89.6
Non-Saudi	66	10.4
Employment status within the first 6 months postpartum		
Employed	132	20.9
Not employed	500	79.1
Household income per month		
<5000 SR	188	29.7
5000–10,000 SR	284	44.9
10,001–15,000 SR	127	20.1
>15,000 SR	33	5.2
Parity		
One child	226	35.8
Two or more children	406	64.2
Mode of birth		
Vaginal	473	74.8
Caesarean	159	25.2
**Fathers**		
Age		
<25 yrs	6	0.9
25–35 yrs	362	57.3
>35 yrs	264	41.8
Education duration		
<12 yrs	378	59.8
≥12 yrs	254	40.2
Nationality		
Saudi	568	89.9
Non-Saudi	64	10.1
Employment status		
Employed	575	91.0
Not employed	57	9.0
**Infants**		
Sex		
Male	338	53.5
Female	294	46.5
Weight at birth		
<2.5 kg	62	9.8
≥2.5 kg	570	90.2
Exclusive breastfeeding at 6 weeks postpartum		
Yes	212	33.5
No	420	66.5

Yrs: years; SR: Saudi Riyal.

**Table 2:** Complementary feeding data by age of introduction (*n* = 632).

Feeding Practices	Age of Introduction of Complementary Foods	
	<17 Weeks *n* (%)	≥17 Weeks *n* (%)
**Total**	395 (62.5)	237 (37.5)
**Explanations given for introducing solid foods**		
Baby hungry	203 (51.4)	118 (49.8)
Baby old enough	105 (26.6)	56 (23.6)
Poor baby weight gain	50 (12.7)	35 (14.8)
Maternal illness	23 (5.8)	22 (9.3)
Other reason	14 (3.5)	6 (2.5)
**Type of food infant was initially given:**		
Baby cereal	244 (61.7)	145 (61.2)
Pureed or mashed fruit or vegetables	77 (19.5)	36 (15.2)
Pureed or mashed meat or fish	33 (8.4)	12 (5.1)
Baby food manufacturers	27 (6.8)	25 (10.5)
Yoghurt	5 (1.3)	11 (4.6)
Other food type	9 (2.3)	8 (3.4)
**Was it difficult to introduce solid foods?**		
Yes	298 (75.4)	115 (48.5)
No	97 (24.6)	122 (51.5)
**Explanations given for difficulty in introducing solid foods**		
Mother uncertain about weaning	34 (8.6)	29 (12.2)
Infant would only accept certain solids and not others	145 (36.7)	112 (47.3)
Infant preferred drinks to food	166 (42.0)	87 (36.7)
Infant experienced choking or vomiting	37 (9.3)	4 (1.7)
Other reason	13 (3.4)	5 (2.1)
**Infant took ≥30 min to finish a meal?**		
Yes	144 (36.5)	56 (23.6)
No	251 (63.5)	181 (76.4)
**Meals a day**		
1	168 (42.5)	35 (14.8)
2	147 (37.2)	138 (58.2)
3 or more	80 (20.3)	64 (27.0)
**Was salt added to the infant’s solid food?**		
Yes	123 (31.1)	143 (60.3)
No	272 (68.9)	94 (39.7)
**Was sugar added to the infant’s solid food?**		
Yes	87 (22.0)	79 (33.3)
No	308 (78.0)	158 (66.7)
**Which foods did mothers avoid giving to their infants?**		
Any poultry, seafood or other meat	227 (57.5)	76 (32.1)
Eggs or other dairy products	104 (26.3)	37 (15.6)
Vegetables (salad)	387 (98.0)	188 (79.3)
Fruit (fresh)	338 (85.6)	201 (84.8)
Nuts	395 (100.0)	237 (100.0)
Honey	387 (98.0)	189 (79.7)
Processed foods	312 (79.0)	175 (73.8)
Chocolate or other sweets	277 (70.1)	107 (45.1)
High salt content foods	86 (21.8)	56 (23.6)
High sugar content foods	157 (39.7)	98 (41.4)
High fat content foods	203 (51.4)	186 (78.5)
Other foods	47 (11.9)	36 (15.2)
**Explanations given for not giving particular foods**		
The infant dislikes them	127 (32.2)	112 (47.3)
Heath reasons (allergies/adverse reactions)	91 (23.0)	23 (9.7)
Food was not cooked at home	47 (11.9)	41 (17.3)
Concern about choking	89 (22.5)	33 (13.9)
Infant is still too young	25 (6.3)	19 (8.0)
Other reason	16 (4.1)	9 (3.8)

**Table 3:** Factors associated with the early introduction of complementary feeding (*n* = 632).

Variables	Age of Introduction of Complementary Foods		Univariate		Multivariate	
	<17 Weeks	≥17 Weeks	OR	95% CI	OR	95% CI
	*n* (%)	*n* (%)				
**Mothers**	395 (62.5%)	237 (37.5%)				
Age						
<25 yrs	81 (53.3)	71 (47.7)	1.17	0.70, 1.95	2.37	1.21, 4.62
25–35 yrs	267 (69.4)	118 (30.6)	2.31	1.46, 3.65	5.27	2.82, 9.86
>35 yrs	47 (49.5)	48 (50.5)	1	Ref	1	Ref
Education duration						
<12 yrs	250 (76.9)	75 (23.1)	3.72	2.65, 5.24	3.88	2.54, 5.93
≥12 yrs	145 (47.2)	162 (52.8)	1	Ref	1	Ref
Nationality						
Saudi	368 (65.0)	198 (35.0)	2.69	1.60, 4.52	2.45	1.23, 4.87
Non-Saudi	27 (40.9)	39 (59.1)	1	Ref	1	Ref
Employment status within the first 6 months postpartum						
Employed	103 (78.0)	29 (22.0)	2.53	1.62, 3.96	6.39	3.53, 11.58
Not employed	292 (58.4)	208 (41.6)	1	Ref	1	Ref
Household income per month						
<5000 SR	113 (60.1)	75 (39.9)	1.42	0.68, 2.98	2.40	0.93, 6.21
5000–10,000 SR	193 (68.0)	91 (32.0)	2.00	0.97, 4.13	3.75	1.53, 9.17
10,001–15, 000 SR	72 (56.7)	55 (43.3)	1.23	0.57, 2.66	1.96	0.80, 4.84
>15,000 SR	17 (51.5)	16 (48.5)	1	Ref	1	Ref
Parity						
One child	149 (65.9)	77 (34.1)	1.26	0.90, 1.77	1.30	0.85, 1.20
Two or more children	246 (60.6)	160 (39.4)	1	Ref	1	Ref
Mode of birth						
Caesarean	139 (87.4)	20 (12.6)	5.89	3.57, 9.74	4.63	2.51, 8.52
Vaginal	256 (54.1)	217 (45.9)	1	Ref	1	Ref
**Infants**	395 (62.5%)	237 (37.5%)				
Sex						
Male	210 (62.1)	128 (37.9)	0.97	0.70, 1.34		
Female	185 (62.9)	109 (37.1)	1	Ref		
Weight at Birth						
<2.5 kg	46 (74.2)	16 (25.8)	1.82	1.01, 3.30	0.49	0.19, 1.27
≥2.5 kg	349 (61.2)	221 (38.8)	1	Ref	1	Ref
Exclusive breastfeeding at 6 weeks postpartum						
Yes	86 (40.6)	126 (59.4)	1	Ref	1	Ref
No	309 (73.6)	111 (26.4)	4.08	2.88, 5.79	3.27	2.15, 4.96

Yrs: years; CI: confidence interval; OR: odds ratio; Ref: reference.
